# Sparse testing designs for optimizing resource allocation in multi‐environment cassava breeding trials

**DOI:** 10.1002/tpg2.20558

**Published:** 2025-02-06

**Authors:** Nelson Lubanga, Beatrice E. Ifie, Reyna Persa, Ibnou Dieng, Ismail Yusuf Rabbi, Diego Jarquin

**Affiliations:** ^1^ Insitute of Biological, Environmental and Rural Sciences Aberystwyth University Aberystwyth UK; ^2^ International Institute of Tropical Agriculture Ibadan Nigeria; ^3^ Agronomy Department University of Florida Gainesville Florida USA

## Abstract

The development of improved cultivars requires establishing multi‐environment trials (METs) to evaluate their performance under a wide range of environmental conditions. However, the high phenotyping costs often limit the capacity to evaluate genotypes in all the target environments. Our main objective was to explore the potential of implementing sparse testing in cassava breeding programs to reduce the cost of phenotyping in METs. The population used in this study consisted of 435 cassava genotypes evaluated in five environments in Nigeria for dry matter (dm) and fresh root yield (fyld). Sparse testing designs were developed based on non‐overlapping (NOL), completely overlapping (OL), and intermediates between NOL and OL genotypes. Three prediction models were assessed (one based on phenotypes only, while two had genomic data). All the three models had a higher predictive ability and a lower mean square error (MSE) when a large training set was used. Predictive ability increased and MSE reduced when genotype‐by‐environment interaction (G × E) was modeled for the same training set sizes and allocations. Predictive ability decreased while MSE increased with the increasing OL genotypes across the environments, suggesting that only a few OL genotypes may be required to set up METs for model training. Sparse testing using a model incorporating G × E could be implemented to reduce cost of phenotyping in cassava METs. If data were available, integrating crop growth models (CGMs) with genomic prediction holds the potential to improve predictive ability. The training population used for sparse testing could be optimized to determine the optimal size and distribution of genotypes to increase the predictive ability and reduce cost under a fixed budget.

**Abbreviations**
G × E: genotype‐by‐environment interaction
GP: genomic prediction
GS: genomic selection
NOL: non‐overlapping
OL: overlapping

## 1 INTRODUCTION

Cassava (*Manihot esculenta*) is a major staple food crop in tropical and subtropical countries. Essential to the cassava breeding program is the reliable and accurate prediction of genotype's performance in multi‐environment trials (METs). Promising genotypes should be evaluated extensively over several years and locations to determine yield performance and stability across environments. In most breeding programs, selections are made at the earlier stages based on information from one or few locations. This implies that genotypes are advanced with little knowledge of the G × E effects and may not be stable in many environments (Jarquín et al., 2017). The presence of G × E in plant breeding is expressed as the change in the relative performance of genotypes across environments, whereby genotypes are ranked in different order across environments. G × E can also result in differences in the magnitude of the response of genotypes to environmental changes. In this case, genotypes maintain their relative ranking across different environments, but the degree of performance differences varies. Assessing G × E is important when evaluating yield under different environments or when targeting stable genotypes with reliable yield under varying conditions (Burgueño et al., 2012; Lopez‐Cruz et al., 2015). The accuracy of predicting superior genotypes is higher when it is based on METs data since multi‐year and multi‐locational trials consist of larger plots with more replicates of each genotype (Cooper & Messina, 2023; Gaffney et al., 2015).

Genomic selection (GS) involves the estimation of genomic estimated breeding values (GEBVs) by summing marker effects that are in linkage disequilibrium (LD) with one or more quantitative trait loci (QTLs) across the entire genome (Bernardo & Yu, 2007). GS uses a prediction model that is first trained using a population of genotyped and phenotyped individuals. The calibrated model is then used to predict GEBVs of selection candidates with genotypic information but no phenotype. The accuracy of the GS models is assessed by using random cross‐validation (CV) methods that split the dataset into training and testing sets. The objective is to use the training set (TRS) (with both genomic and phenotype data) to predict the phenotypes of the testing set (with only genomic data). The predictive ability is estimated by correlating the GEBVs with the actual phenotypic information of the testing set (Meuwissen et al., 2001). GS is currently implemented in many crops and trees; for example, Messina et al. (2023) provide a summary of 20 years of GS in drought tolerance in maize. Lubanga et al. (2023) showed GS strategies that can be implemented in tea breeding programs. Isik (2022) presented an overview of the GS progress in forest tree species. Bassi et al. (2016) showed practical guidelines and breeding schemes for implementing GS in wheat.

Evaluating all genotypes in all environments may not be feasible due to limited resource availability, but more importantly, it may not be desirable due to low efficiency of resource use. Using genomic data and observing a reduced number of genotype‐in‐environment combination offer the possibility of assessing the marker alleles in all environments as well as the marker × environment interactions. Therefore, it is possible to make inferences on performance of unobserved genotypes. The information of the response patterns of the markers and the marker × environment interactions can be leveraged to improve the predictive ability of the untested genotypes (Burgueño et al., 2012; Cooper et al., 2014; Lopez‐Cruz et al., 2015).

Genome‐enabled sparse testing is a prediction strategy that attempts to optimize the allocation of resources in which the phenotypic information of lines/genotypes is split across environments (Jarquin et al., 2020). Sparse testing using genomic prediction (GP) is conducted by combining different CV schemes and models incorporating genomic information as main and interaction G × E effects to predict the non‐observed genotype‐in‐environment combinations. The best way to benefit from sparse testing is to establish MET that are cost‐effective, without negatively impacting the selection accuracy of genotypes in breeding trials. The accuracy of predicting unobserved genotypes in METs is influenced by (a) the number of overlapping (OL) genotypes across environments (training composition), (b) the number of environments where each genotype is tested, (c) the implemented prediction model, and (d) the number of phenotypes per genotype (Garcia‐Abadillo et al., 2024; Jarquin et al., 2020).

To our knowledge, there are no studies incorporating genome‐enabled sparse testing in cassava breeding programs. The aims of this study were to (1) test the feasibility of using genome‐enhanced sparse testing to establish cost‐effective multi‐environment cassava trials, and (2) assess the prediction accuracy of three models in different sparse testing designs for two traits using a fixed number of genotypes as a testing set (∼80%) in each environment.

## 2 MATERIALS AND METHODS

### 2.1 Phenotypic dataset

A total of 435 genotypes at the International Institute of Tropical Agriculture were evaluated at three diverse sites in Nigeria. Field trial evaluation at Ikenne (Derived Savannah, 6° 52ʹ 12.00ʺ N, 3° 42ʹ 36.00ʺ E) was done in 2020 and 2021, Mokwa (Southern Guinea Savannah, 9° 17ʹ 41.35ʺ N, 5° 03ʹ 14.83ʺ E) in 2022, and Onne (Humid Forest, 4° 43ʹ 25.74ʺ N, 7° 9ʹ 5.82ʺ E) in 2022 and 2023. The trials were planted in an alpha‐lattice design with two replications. Each plot consisted of two rows of five plants with spacing between and within rows of 1 and 0.8 m, respectively.

### 2.2 Phenotyping for fyld and dm

All the plant genotypes were harvested at 12 months after planting and fresh root yield (fyld) weight was recorded in kilograms. Six cassava roots from each genotype were randomly selected from each plot and crushed. A subsample of 100 g was then removed from the crushed cassava and dried constantly at 80°C for 48 h. The resulting weight was recorded as dry matter (dm).

### 2.3 SNP genotyping and quality control

DNA extraction was performed at the Diversity Arrays Technology (DArT) facility (https://www.diversityarrays.com/orderinstructions/plant‐dna‐extraction‐protocol‐for‐dart/). The DNA quality of each sample was qualitatively and quantitatively assessed on 0.8% agarose gel and Nanodrop 2000c spectrophotometer (Thermo Scientific), respectively. Genotyping was done using the DarTseqLD methodology (https://www.diversityarrays.com/technology‐and‐resources/dartseq/).

**Core ideas**

- Sparse testing designs could significantly reduce phenotyping cost of multi‐environment trials (METs) in cassava breeding programs.
- The model incorporating genotype‐by‐environment interaction (G × E) consistently had high predictive ability and the low mean square error (MSE) across all sparse testing designs.
- Predictive ability increased while the MSE decreased with an increasing number of non‐overlapping genotypes across environments.

After quality control, 3002 single nucleotide polymorphisms (SNPs) were obtained. The SNPs were converted to numeric allele classes (0, 1, 2) for homozygous minor, heterozygous, and homozygous major alleles, respectively, using Plink 1.9 (Purcell et al., 2007). Further filtering involved removing all markers with more than 20% missing values and a minor allele frequency below 5%. SNPs with a call rate lower than 80% and heterozygosity larger than 95% were also excluded. The final set of genotypes contained 2984 markers. Mean imputation was applied to the missing markers.

### 2.4 Phenotypic analysis

The initial process of developing sparse testing design assumes that all genotypes are evaluated in all environments. Therefore only those genotypes that met this requirement were selected for this study. Out of the 451 initial genotypes, 435 were selected for analysis since these were observed across all the five environments. The phenotypic analyses were performed in two stages. The first stage involved calculating the best linear unbiased predictors (BLUPs) of each genotype at each environment (site × year combination). The calculation of BLUPs at each environment in the first stage was performed using ASReml‐R Version 4.2 (Butler et al., 2023) in R statistical programming language (R Core Team, 2021) using the following model (Equation 1):

(1) $$y_{ijk}=\mu +L_{i}+R_{j}+B_{k}{\left(R\right)}_{j}+e_{ijk}$$

where y *_ijk_* represents the observed phenotype, *μ* is the overall mean, *L_i_* is the random effect of the *i*th genotype assuming L *_i_* $\;\overset{iid}{∼}N\left(0,\sigma_{L}^{2}\right)$, and $\sigma_{L}^{2}$ is the variance component of the genotypes; *R_j_* is the fixed effect of the *j*th replicate, $B_{k}{\left(R\right)}_{j}\;$ is the random effect of the *k*th incomplete block nested within the *j*th complete replication, such that $B_{k}{\left(R\right)}_{j}\;\overset{iid}{∼}N\left(0,\sigma_{BR}^{2}\right)$, and $\sigma_{BR}^{2}$ is the variance component of the block effect nested within replication; $e_{ijk}$ is the random error term with $e_{ijk}\overset{iid}{∼}N\left(0,\sigma_{e}^{2}\right)$ and $\sigma_{e}^{2}$ is the error variance addressing the non‐explained variability by the previous model terms.

The broad‐sense heritability (*H* ^2^) at each environment was calculated on an entry‐mean basis and genotypic and error variances were extracted from Equation (1). *H* ^2^ was estimated as shown in Equation (2).

(2) $$H^{2}=\frac{\sigma_{g}^{2}}{\sigma_{g}^{2}+\sigma_{e/r}^{2}\;\;\;\;}$$

where $\sigma_{g}^{2}$ is genotypic variance, $\sigma_{e}^{2}$ is error variance, and *r* is the number of replicates within each environment.

### 2.5 Prediction models

The second stage of the analysis involved using the BLUPs obtained in the first stage (Equation 1) to fit three different prediction models described below in Equations (3), (5), and (6). Then, the predictive ability and mean square error (MSE) of the three models for the different TRS sizes and allocation designs were computed within each environment.

#### 2.5.1 Environment + Genotype (E + L)

In this model, the response variable corresponds to the adjusted phenotypes (*y_il_*) (BLUPs) obtained after fitting the corresponding mixed linear model as previously described in Equation (1). Consider that *y_il_* represents the response of the *i*th genotype at the *l*th environment. In this case, the environment and genotype effects are considered random outcomes:

(3) $$y_{il}=\mu +E_{l}+L_{i}+e_{il}$$

where *E_l_* is the random effect of the *l*th environment such that E_l_ $\;\overset{iid}{∼}\;N\left(0,\sigma_{E}^{2}\right)$, and $\sigma_{E}^{2}$ is the variance component of the environment effect, *L_i_* is the random effect of the *i*th genotype assuming L_i_ $\;\overset{iid}{∼}\;N\left(0,\sigma_{L}^{2}\right)$, where $\sigma_{L}^{2}$ is the variance of the genotype effect, and *e_il_* is the random error term, such that e_il_ $\;\overset{iid}{∼}\;N\left(0,\sigma_{e}^{2}\right)$, where $\sigma_{e}^{2}$ is the error variance.

#### 2.5.2 Environment, genotype, and genomic main effects (E + L + G)

This model is an extension of M1 (Equation 3). It considers the inclusion of the genomic random effect of the *i*th genotype $g_{i}$, which approximates its genetic value using molecular marker information. This model component can be defined by the regression on *p* marker covariates:

(4) $$g_{i}=\sum_{m=1}^{p}x_{im}b_{m}$$

where $x_{im}$ is the genomic information of the *i*th genotype at the *m*th marker, and $\;b_{m}$ is the corresponding marker effect, assuming b *_m_* $\overset{iid}{∼}N\left(0,\sigma_{b}^{2}\right)$, and with $\;\sigma_{b}^{2}$ being the corresponding variance component which is homogenous to all markers. The vector of genomic effects $g=\{g_{i}\}$ follows a multivariate normal density with zero mean and variance‐covariance matrix:

$$\mathrm{cov}\left(g\right)=G\sigma_{g}^{2}\;\text{such that}\;g∼N\left(0,G\sigma_{g}^{2}\right)$$

where $G\;\alpha \frac{{\mathrm{XX}}^{\prime }}{p}$ is the genomic relationship matrix, **X** is the centered and standardized (by columns) matrix of molecular markers, and $\sigma_{g}^{2}\;\alpha \;\sigma_{b}^{2}$ is the genomic variance. The resulting model is given as:

(5) $$y_{il}=\mu +E_{l}+L_{i}+g_{i}+e_{il}$$

where the genotype effects of the vector of genomic random effects g are correlated such that M2 allows the borrowing of information across lines. A disadvantage of this model is that upon an environmental constant, the GEBV for a genotype tested in different environments is the same, regardless of the environment. To allow a specific genomic effect in each environment, the interaction term (G × E) is included in model M3 below.

#### 2.5.3 Environment, genotype, genomic, and genomic × environment interaction effects (E + L + G + G × E)

This model is an extension of model 2 (Equation 5) by adding the interaction between the markers and environments ($g\times E_{il})$ via covariance structures as shown by Jarquín et al. (2014). The reaction norm model is an extension of the random effect genomic best linear unbiased predictor model where the interactions between each SNP and each environment are indirectly modeled using the following linear predictor:

(6) $$y_{il}=\mu +E_{l}+L_{i}+g_{i}+g\times E_{il}+e_{il}$$

where the $g\times E_{il}$ term corresponds to the interaction between the genetic value of the *i*th genotype and the *l*th environment. This interaction term is assumed to follow a multivariate normal distribution such that 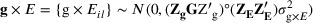 where $Z_{g}$ and $Z_{E}$ are the incidence matrices for connecting phenotypes with genotypes and environments, respectively, $\sigma_{g\times E}^{2}$ is the variance component associated to g×E, and “°” represents the Hadamard product between two matrices. The Hadamard product is used to model the covariance structure of interactions. It is the cell‐by‐cell product of two covariance structures, one describing the genetic information and the other describing the environmental effects.

### 2.6 Training population and sparse testing designs

The following sparse testing designs were developed in this study: (a) nonoverlapping (NOL) genotypes between environments (i.e., all genotypes are tested in different environments and observed only once across environments), (b) genotypes completely OL across environments (i.e., a set of genotypes are field evaluated in all the environments), and (c) varying numbers of different NOL/OL genotypes. OL genotypes incorporate reluctance while NOL genotypes generate diversity in the sampling of haplotypes.

A total of 435 genotypes were evaluated in five environments (site × year combination). We constructed different sparse testing designs by systematically combining NOL and OL genotypes across environments. Initially, five sets of NOL genotypes, that is, 87 genotypes in each set (435 genotypes divided by 5) were randomly generated. These NOL sets of genotypes were assigned to each of the five environments (Table 1). This was the initial allocation of only the unique genotypes to the largest TRS size (i.e., 87) across environments, creating an 87/0 sparse testing design (i.e., 87 NOL and 0 OL genotypes). The goal was to predict the remaining 348 genotypes within each environment (435 − 87 = 348). In this way, the 435 genotypes were observed once across the five environments. Sequentially, within each environment, 10 genotypes out of the 87 genotypes observed in each environment were randomly selected and masked as missing values (10 × 5 = 50). From the 50 genotypes masked as missing values across environments, a set of 10 genotypes was selected and marked as observed in all the five environments. This implied that 10 common genotypes were observed in all the five environments while 77 unique genotypes were observed within each environment. This means that out of the initial 435 unique genotypes, only 395 (435 − 50 + 10) were observed at least once and 40 were not observed at all. Therefore, the total number of plots observed was 10 × 5 (common in the five environments) + 5 × 77 (unique genotypes in each of the five environments) = 435. The calibration set consisted of all the 435 genotype‐in‐environment combinations, while across environments, the prediction set consists of the remaining 435 × 4 combinations. The different NOL/OL designs were gradually reduced by sets of 10 genotypes from 87 to 7 and then 0 (87, 77, 67, …, 17, 7, 0) as shown in Table 1. The complete OL designs include 0/87, 0/77, 0/67, 0/57, and 0/47 (Table 1).

**TABLE 1:** Training set sizes and the allocation designs (nonoverlapping [NOL] and overlapping [OL]) of cassava genotypes observed within each environment.

Training set sizes	Allocation designs (NOL/ OL)								
87	87/0	77/10	67/20	57/30	47/40	37/50	27/60	17/70	7/80 0/87
77	–	77/0	67/10	57/20	47/30	37/40	27/50	17/60	7/70 0/77
67	–	–	67/0	57/10	47/20	37/30	27/40	17/50	7/60 0/67
57	–	–	–	57/0	47/10	37/20	27/30	17/40	7/50 0/57
47	–	–	–	–	47/0	37/10	27/20	17/30	7/40 0/47

*Note*: The first column on the left shows the training set sizes, while the remaining columns represent the allocation designs.

In addition, the within‐environments TRS sizes were also gradually reduced by sets of 10 genotypes from 87 to 47. While reducing the TRS sizes gradually, precaution was taken to allow the different compositions between NOL and OL genotypes across environments. Combinations of NOL/OL allocation strategies for different TRSs are shown in Table 1.

### 2.7 Model assessment via cross‐validation schemes

The TRS compositions represent two of the most common CV strategies used in GP studies (CV2 and CV1). CV2 strategy is mostly applicable when predicting incomplete field trials where some genotypes have been observed in some environments but not in others (e.g., incomplete field trials). CV1 strategy is mainly used to predict the performance of newly developed genotypes that have not been observed in any of the tested environments, but there is available phenotypic information for other genotypes in these environments. In our study, CV1 was represented by 0/87, 0/77, 0/67, 0/57, and 0/47 allocation designs, which comprised NOL and zero OL in each environment. The remaining allocation designs were assigned to CV2 (see Table 1). These CV schemes were used to assess the model's predictive ability on a trial basis using the Pearson's correlation and the MSE between predicted and observed values within each environment. The smaller the value of MSE, the higher accuracy of the model. It was estimated as the mean of the squared deviations between observed and predicted values in the testing set.

$$\mathrm{MSE}=\frac{1}{n}\sum_{i=1}^{n}{\left({\hat{y}}_{i}-y_{i}\right)}^{2}$$

where *n* = 384 represents the number of cassava genotypes in each within‐environment testing set, ${\hat{y}}_{i}$ and $y_{i}$ are the predicted and observed values of the *i*th genotype at the current environment. The final MSE values were the average of 10 replicates.

Initially, the within‐environment TRSs were composed of 20% (87 genotypes), while the prediction sets represented the remaining 80% (348 genotypes). The within‐environment TRSs were combined and used for predicting the testing sets that were not observed in each environment. In all the cases, the testing set was composed of 348 genotypes within each environment. Thus, the size of the prediction set was always constant. This process was repeated 10 times, and the correlations between the predicted and observed values within the same environment were computed for all environments. The GP models were fitted using the BGLR package (Pérez & de los Campos, 2014).

## 3 RESULTS

### 3.1 Phenotypic summary and broad‐sense heritability

**FIGURE 1:**
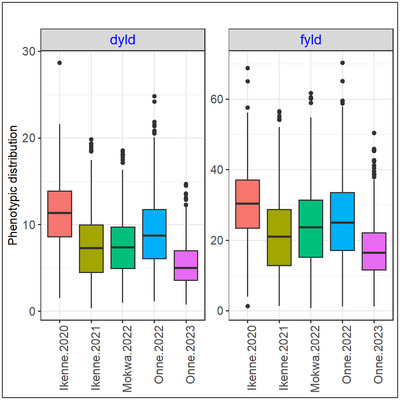
Box plot showing the distribution of dry matter (dm) and fresh root yield (fyld) in the five environments.

Mean phenotypic values, standard deviations (SDs), and broad‐sense heritabilities (*H* ^2^) are provided for dm and fyld under the five environments (Table 2). Large fluctuation was observed for the two traits in all the five environments (Figure 1). Mean phenotypic values for dm ranged from 29.84 to 37.31 g for Onne.2023 and Ikenne.2020, respectively. Similarly, mean fyld ranged from 17.21 to 30.44 kg for Onne.2023 and Ikenne.2023, respectively. There was a large difference between site and small between year variation for the phenotypic values across the five environments. This highlights the impact of the environment on dm and fyld production. The average SD was rather stable across environments, ranging from 2.19 to 2.75 for dm and 5.16 to 6.96 for fyld, showing a reliable level of precision in the estimation. Broad‐sense heritability was moderate ranging from 0.80 to 0.87 for dm and 0.61 to 0.78 for fyld (Table 2).

**TABLE 2:** Summary of the mean phenotypic values for dry matter (dm) and fresh root yield (fyld) in the five environments (site by year combination). The number of genotypes obtained from the International Institute of Tropical Agriculture (IITA) multi‐environment trial (MET) at the advanced yield testing stage (2020–2023) is shown.

Site	Year	Environment	Genotypes	dm			fyld		
				Mean (g)	SD	*H* ^2^	Mean (kg)	SD	*H* ^2^
Ikenne	2020	Ikenne.2020	435	37.31	2.75	0.80	30.44	6.37	0.78
Ikenne	2021	Ikenne.2021	435	34.04	2.72	0.80	21.41	6.96	0.78
Mokwa	2022	Mokwa.2022	435	30.52	2.19	0.81	23.49	5.79	0.61
Onne	2022	Onne.2022	435	33.60	2.40	0.87	25.74	6.52	0.73
Onne	2023	Onne.2023	435	29.84	2.40	0.87	17.21	5.16	0.73

*Note*: The mean dm and fyld for each environment are the BLUPs. Environment column corresponds to the unique site by year combination that constitutes a single environment, for which there are five in this study. The broad‐sense heritabilities (*H* ^2^) and standard deviation (SD) in each environment are also shown for each trait.

### 3.2 Genomic predictive ability and MSE under different sparse testing allocation designs

**FIGURE 2:**
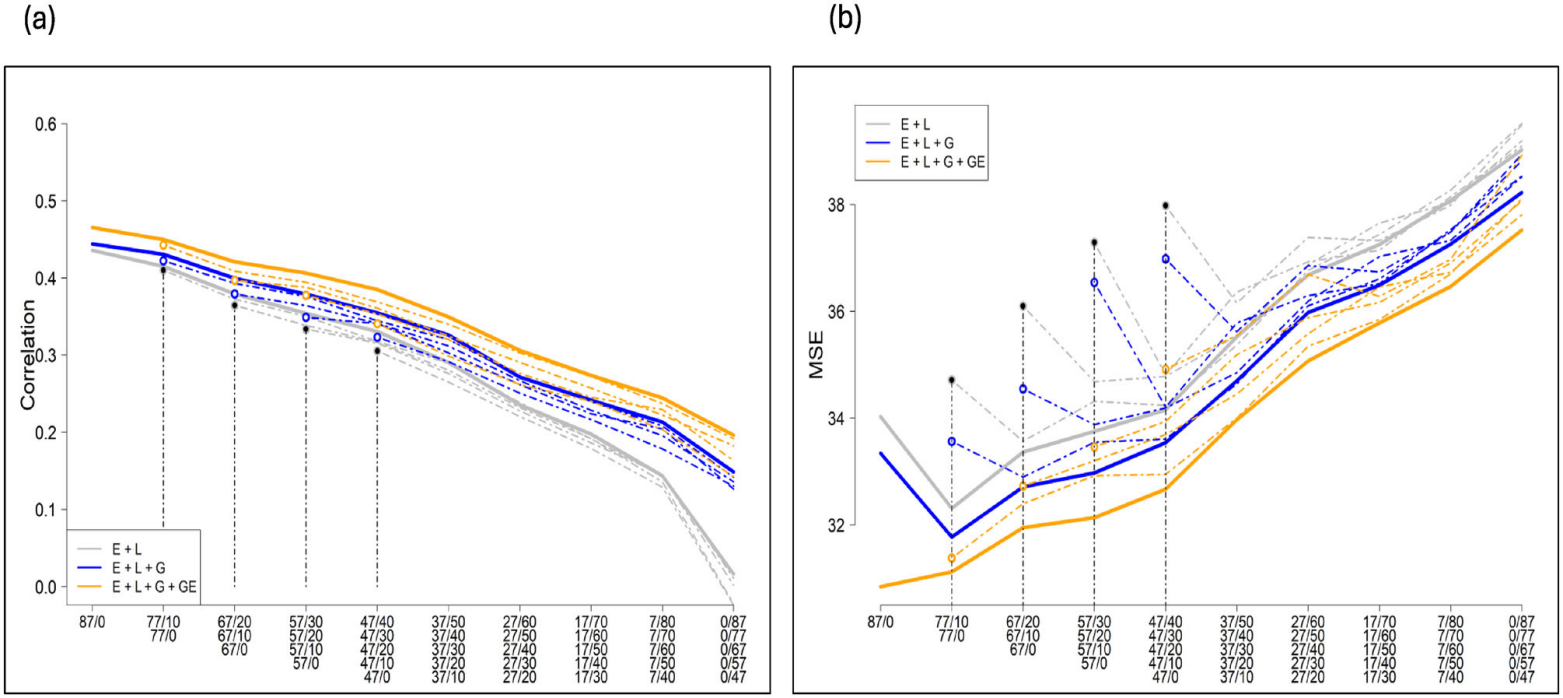
Predictive ability (a) and mean square error (MSE) (b) for fresh root yield (fyld) under different sparse testing designs. The gray line represents M1, blue line M2, and orange line M3. The solid and dashed‐dotted lines represent the mean for different sizes and compositions of the allocation designs.

We studied the predictive ability and MSE of three models under different calibration sets (TRS sizes and compositions). A summary of the results of the predictive ability and MSE for fyld and dm are presented in Figures 1 and 2, respectively. The plots show the average (10 replicates) mean predictive ability and MSE across the five environments for different TRS sizes and compositions consisting of NOL and OL cassava genotypes for the three prediction models: M1, M2, and M3. The solid thick line represents the mean average predictive ability corresponding to the largest within‐environments TRS size (87 genotypes), while the thin dashed lines correspond to the reduced TRS sizes (77–47). The starting point on the left side of the lines represents the mean average for the scenarios where all the genotypes were observed only once across environments (87, 77, 67, 57, and 47), while the other extreme of the lines (right side) corresponds to the case where all of the genotypes (e.g., 87 out of 87, 77 out of 77, 67 out of 67, 57 out of 57, and 47 out of 47) are common across environments. The number of common genotypes increased by 10 moving from the left to the right of the plots. Individual results for each model and trait can be obtained in Tables S1–S12.

**FIGURE 3:**
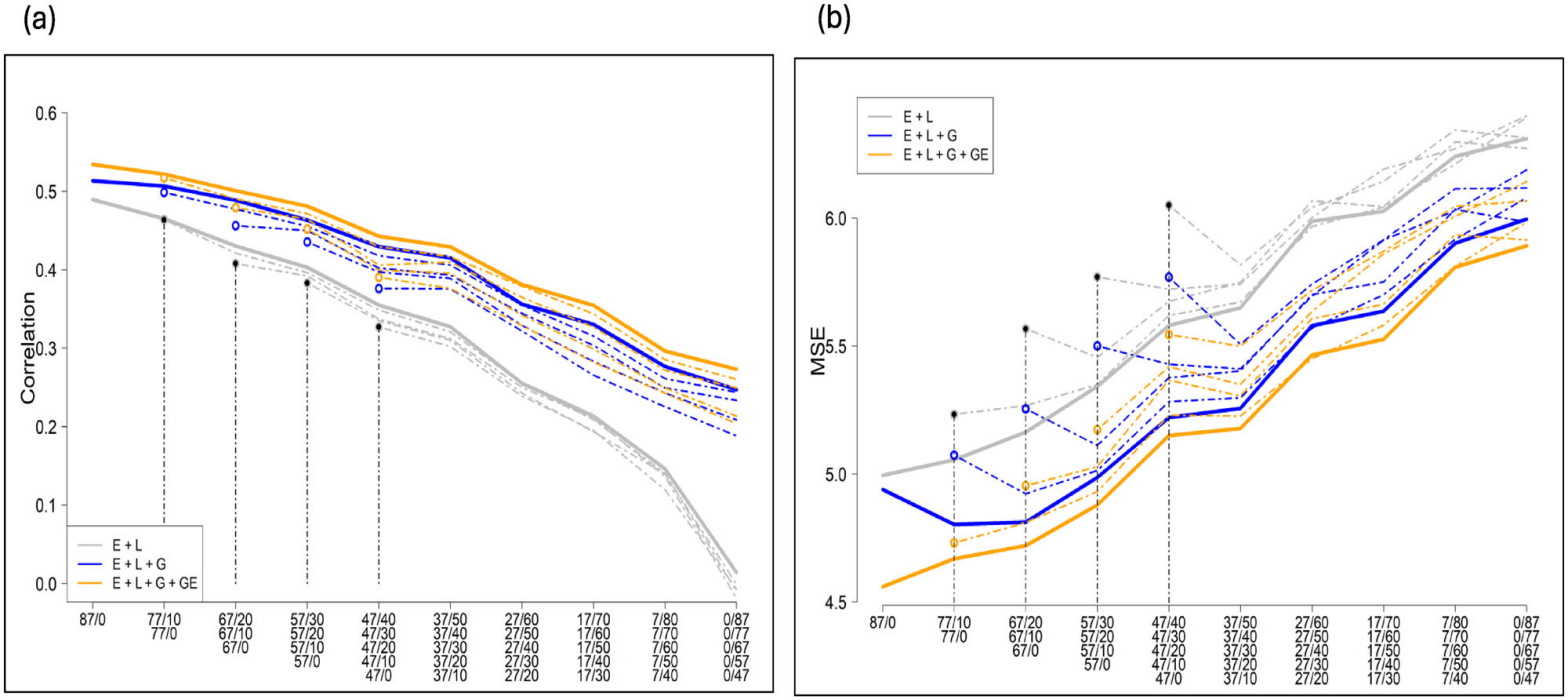
Predictive ability (a) and mean square error (MSE) (b) for dry matter (dm) under different sparse testing designs. The gray line represents the M1, blue line the M2, and orange line the M3. The solid and dashed‐dotted lines represent the mean for different sizes and compositions of the allocation design.

In general, we observed a similar trend in predictive ability and MSE for all traits. The predictive ability for all the models, M1, M2, and M3 decreased as the number of OL genotypes was increased across the environments. The highest predictive ability was recorded under the completely NOL scenario, while it was the lowest when all the genotypes were OL for both traits (Figures 2a and 3a). The model M3 showed the highest predictive ability, followed by M2, while M1 had the lowest across the allocation designs for both traits (Figures 2a and 3a). The predictive ability ranged from 0.47 to −0.02 for fyld (Tables S4 and S6) and 0.53 to −0.02 for dm (Tables S1 and S3).

The MSE increased gradually as the number of OL genotypes increased for both traits (Figures 2b and 3b). The highest MSE was reported in the completely OL scenario, while the lowest MSE was recorded under the NOL strategy. In all the allocation designs, the model M3 had the lowest MSE, while M1 had the highest in both traits. The MSE ranged from 4.56 to 6.31 for dm (Tables S7 and S9) and 30.85 to 39.49 for fyld (Tables S10 and S12). Additionally, the results showed that all the three models had a higher predictive ability and a lower MSE when a large TRS was used (Figures 2b and 3b). Predictive ability improved and MSE reduced when G × E was modeled.

## 4 DISCUSSION

Previous work has demonstrated the potential of applying GP to identify superior genotypes with high breeding values in cassava breeding programs (de Andrade et al., 2019; Jose‐Santhi & Singh, 2023; Okeke et al., 2017). Here we build upon these findings by implementing sparse testing that could enable increasing the size of the breeding program and thus hasten the much‐needed genetic gain in cassava. Three different models under different TRS sizes and compositions using OL and NOL sets of genotypes were considered. We demonstrate that cassava breeding program could benefit from increased breeding efficiency and reduced cost of phenotyping by implementing sparse testing with a model incorporating G × E in METs.

### 4.1 G × E in cassava

Our study showed large variations in dm and fyld across the five environments, demonstrating the influence of environmental factors and gene–environment interactions on cassava productivity. Tumuhimbise et al. (2014) and Mbe et al. (2024) showed large variations in average early fresh storage root yield across locations and years. These fluctuations in yield of cassava highlight the dynamic nature of crop growth systems and emphasize the need for approaches that effectively account for G × E to improve yield predictions. In our study, dm and fyld had varying mean phenotypic values in the different environments, shedding light on the genetic control and sensitivity to environmental influences of these two traits. Additionally, the large G × E variance (Figure S1) observed in our study implies that G × E is an important determinant of the performance and needs to be accounted for when predicting cassava in METs. The performance of the cassava genotypes could have been influenced by the unique environmental factors such as rainfall, temperature, and soil composition found in these environments. The differences in the performance of the cassava genotypes in the five environments could be explained by the large changes in the substitution effects of the QTL involved in the trait, promoted by a change in the environment (Powell et al., 2021).

### 4.2 Predictive ability and MSE under different allocation designs

Genome‐enabled sparse testing has a huge potential in plant breeding because it could reduce the overall phenotyping cost of METs by (a) increasing the number of tested genotypes and environments for a fixed budget (testing capacity) or (b) reducing phenotyping costs for given target sets of genotypes and environments (Jarquin et al., 2020). The slightly higher predictive ability values observed by M3 are similar to results reported by Persa et al. (2023) in soybean, Crespo‐Herrera et al. (2021) in wheat, and Garcia‐Abadillo et al. (2024) in sugarcane. Including G × E component in model M3 allows the borrowing of information from related genotypes tested in other environments (Jarquín et al., 2014). This enables the prediction of unobserved genotypes in different environments; hence, it could result in substantial savings in phenotyping costs. Modeling covariance matrices to account for G × E allows the use of information from correlated environments (Burgueño et al., 2012). Mixed models that allow the incorporation of a genetic covariance matrix calculated from marker data, rather than assuming independence among genotypes, could improve the estimation of the genetic effects (Jarquín et al., 2014). The benefit of using genetic covariance matrices in G × E mixed models is that the model relates genotypes across environments even when the genotypes are not present in all environments (Monteverde et al., 2018). Therefore, accounting for G × E in METs trials could improve prediction accuracy as reported by Jarquín et al. (2017) and Sukumaran et al. (2018) in wheat and Mageto et al. (2020) in maize.

In this study, predictive ability decreased as the number of OL genotypes was increased across the environments. This implied that all the models had the highest predictive ability when more diverse sets of genotypes were observed across environments. By contrast, the MSE increased as the number of common genotypes increased across the environments. The MSE was used to evaluate the performance of the three models, whereby smaller values indicated higher accuracy of the model. The high predictive ability and low MSE values obtained under the NOL scenario suggest that the cassava breeding program does not require testing a large set of common genotypes across environments. This could be advantageous in cassava breeding program due to the constraints on the land and seeds/material availability. Persa et al. (2023) reported an increase in predictive ability in soybean when 10 genotypes were overlapped across all the environments. They used a much larger TRS size (ranging from 95 to 195 genotypes per environment for predicting 1560 genotypes at each environment). Similarly, Crespo‐Herrera et al. (2021) showed that an OL set of 30–50 wheat lines provided stable predictive ability in all the environments. Our results showed a similar trend to Garcia‐Abadillo et al. (2024), who implemented sparse testing designs in sugarcane.

Reducing the size of the training population resulted in decreased predictive ability in all the allocation designs. Persa et al. (2023) and Garcia‐Abadillo et al. (2024) also showed that predictive ability reduced with a decreased TRS size across all the allocation designs. These results emphasize the importance of large TRS sizes for a successful genome‐enabled sparse testing in METs. Several studies have reported the importance of a large training population in GP. For instance, according to Asoro et al. (2011), the prediction accuracy increased as the number of markers and training size became large in North American oats. Neyhart et al. (2017) evaluated several methods of updating the training population in a long‐term GS program and reported that using a smaller but more recent training population provided a slight advantage in prediction accuracy and genetic gain.

### 4.3 Implementation of sparse testing in cassava breeding program

Initially, 20% of the population (87 genotypes) was selected as the TRS, while the remaining 80% (348 genotypes) were the testing set in each of the five environments. The TRS was used for predicting the unobserved genotypes (the testing set) in each environment (Section 2.6). We observed that maintaining 20% of the phenotypic records in the multi‐environment TRS achieved a moderate predictive ability. This suggests that the cost of phenotyping genotypes in METs could be reduced by up to 80% since only 20% are phenotyped. Similarly, the testing capacity could be increased fivefold for a fixed budget.

The greatest bottleneck in plant breeding programs is evaluating promising genotypes in the field due to the high cost involved in setting up METs (Crossa et al., 2017). Currently, the high cost of field evaluation at different locations and years makes phenotyping the most important constraint in plant breeding programs. Allocation of resources using sparse testing could reduce the cost of breeding and increase the number of genotypes evaluated in METs while maintaining a high selection accuracy. Sparse testing involves altering the original multi‐environment breeding trial system into a system where not all genotypes are tested in all environments. The variability of the environments of interest can be captured through a reduced set of genotypes tested in each of these environments, which can then be used to predict the untested genotypes. This reduces the costs of acquiring more land to test more genotypes. Observing few unique genotypes in all the environments makes it possible to estimate marker alleles in all environments of interest and the marker × environment interaction. The response patterns of the markers and the marker × environment interaction are used to enhance the genomic predictive ability of the unobserved individuals in the environments. In this study, we implemented the reaction norm model (Jarquín et al., 2014), which accounts for the interaction between molecular markers and the environment through a covariance structure. Incorporating G × E in GP improves the selection accuracy when calculating breeding values of genotypes in different environments (Heslot et al., 2014).

### 4.4 Integrating crop growth models with whole genome prediction

Significant genotype‐by‐environment‐by‐management (G × E × M) interactions are a major challenge in plant breeding programs (Messina & Cooper, 2023). This challenge is especially true for a crop like cassava that is often grown in diverse environments and management conditions across Asia and Africa, leading to fluctuations in yield. GP models estimate breeding values based on quantitative genetic theory and are statistical representations of a complex biological system. The use of GP by incorporating G × E term has been shown to increase the predictive ability in METs (Heslot et al., 2014). However, these statistical models do not explicitly take into consideration most of the biology that contributes to G × E (Hammer et al., 2006). Crop growth model (CGM) predicts multiple phenotypes for cultivars across a wide range of METs by simulating the daily growth of the plant in each environment from sowing to maturity (Cooper et al., 2016). CGMs require large multi‐environment phenotypic datasets for each cultivar to calibrate the estimation of the genotype‐specific parameters (GSPs) that interact with the weather, management, and site characterization inputs to produce the observed phenotypes (Washburn et al., 2020). At present, CGMs are increasingly being used in plant breeding to expand the inference of evaluations of breeding germplasm to environments that have not been tested and to select for adaptation to future climates. Okoma et al. (2024) assessed the feasibility of using a large‐scale CGM calibration in cassava breeding program. They concluded that CGM calibration could become a routine component of the cassava breeding data analysis cycle. Technow et al. (2015) proposed a method of integrating CGM with whole genome prediction (WGP) through approximate Bayesian computation that is CGM–WGP. The CGM–WGP methodology uses a CGM as part of the calculation of the likelihood function step in the standard WGP algorithm (Technow et al., 2015). Messina et al. (2018) used the CGM–WGP methodology to train models using data from multiple environments to evaluate whether the CGM–WGP methodology can enable improved phenotypic prediction when G × E is an important determinant of performance in maize. They improved the CGM–WGP methodology by reformulating the model as a Bayesian generalized linear hierarchical model and sampling the posterior distribution using a Metropolis‐within‐Gibbs sampling algorithm. This increased the efficiency of the algorithm that enabled the use of multiple environments and larger populations than those used in previous studies in maize. Messina et al. (2022) further developed a framework based on the CGM–WGP system, which is enabled by advanced phenomics and the integration of symbolic and sub‐symbolic artificial intelligence. Diepenbrock et al. (2022) showed that CGM–WGP had a higher prediction accuracy compared to BayesA for untested genotypes evaluated in untested environments. Jighly et al. (2023) used a CGM–WGP model to predict the performance of new wheat genotypes for phenology, nitrogen, and biomass traits. They reported that the CGM–WGP model simulated more heritable GSPs compared with the CGM and gave smaller errors for the observed phenotypes.

These results suggest that more efforts could be put into utilizing CGM–WGP models to improve the predictive ability and identify new phenotypes adapted to climate change in breeding programs (Messina & Cooper, 2023). Additionally, Jighly et al. (2023) suggested that future efforts should focus on calibrating CGM–WGP models using high‐throughput phenotypic measurements that are cheaper and less laborious.

### 4.5 Training population optimization for phenotyping

The design of the TRS is one of the key steps in the implementation of GS in plant breeding programs (Heslot & Feoktistov, 2020). The size of the TRS used in GS affects the accuracy of GP models and correlates positively with the increase in size (Norman et al., 2018). However, Fernández‐González et al. (2023) showed a plateau in prediction accuracy increment after reaching an optimum TRS size. Increasing the size of TRS is expensive since more genotypes are required for phenotyping (Isidro y Sánchez & Akdemir, 2021). It is important to determine the optimal distribution of genotypes in METs to achieve the best balance between the number of genotypes tested in the field and the predictive ability of GS models to maximize the selection gain under a fixed budget (Jarquin et al., 2020).

The adoption of strategies for optimizing the TRS could increase the genetic gains and improve breeding efficiency by (i) decreasing the number of genotypes to be tested and, therefore, reducing phenotyping cost and time (Isidro y Sánchez & Akdemir, 2021), (ii) increasing the prediction accuracies of unobserved genotypes (Costa‐Neto et al., 2021), and (iii) improving the efficiency of resource allocation (Alemu et al., 2024). TRS optimization methods with the capability to automatically find the optimal TRS size have been recently developed and can be used practically in breeding programs (Akdemir et al., 2021; Wu et al., 2023).

## 5 CONCLUSION

Most commercial plant breeding programs are implementing GS due to the reduced cost and availability of molecular markers. However, it is expensive to evaluate genotypes in different environments. We compared three models and investigated the potential of implementing sparse testing in cassava breeding program. Model M3 that incorporated G × E had a higher predictive ability and reduced MSE values than the main effect models M1 and M2 for the same allocation designs. Predictive ability decreased as common genotypes (OL) increased across the environments. This could suggest that high predictive ability does not require testing a large set of common genotypes across environments, which could be valuable in cassava breeding program to reduce the cost of field evaluation. The training population used for sparse testing could be optimized to determine the optimal distribution and size of genotypes to increase the predictive ability and reduce cost under a fixed budget. CGM could be integrated with WGP to improve the predictive ability.

## AUTHOR CONTRIBUTIONS

**Nelson Lubanga**: Conceptualization; data curation; formal analysis; investigation; methodology; project administration; software; validation; visualization; writing—original draft; writing—review and editing. **Beatrice E. Ifie**: Writing—review and editing. **Reyna Persa**: Data curation; formal analysis; validation. **Ibnou Dieng**: Methodology; writing—review and editing. **Ismail Yusuf Rabbi**: Funding acquisition; project administration; resources; writing—original draft; writing—review and editing. **Diego Jarquin**: Conceptualization; data curation; formal analysis; resources; software; supervision; validation; visualization; writing—original draft; writing—review and editing.

## CONFLICT OF INTEREST STATEMENT

The authors declare no conflicts of interest.

## Supporting information




**Table S1**. Predictive ability of the M1 model for dm under different sparse testing designs
**Table S2**. Predictive ability of the M2 model for dm under different sparse testing designs.
**Table S3**. Predictive ability of the M3 model for dm under different sparse testing designs.
**Table S4**. Predictive ability of the M1 model for fyld under different sparse testing designs
**Table S5**. Predictive ability of the M2 model for fyld under different sparse testing designs.
**Table S6**. Predictive ability of the M3 model for fyld under different sparse testing designs.
**Table S7**. MSE values of the M1 model for dm under different sparse testing designs.
**Table S8**. MSE values of the M2 model for dm under different sparse testing designs.
**Table S9**. MSE values of the M3 model for dm under different sparse testing designs.
**Table S10**. MSE values of the M1 model for fyld under different sparse testing designs.
**Table S11**. MSE values of the M2 model for fyld under different sparse testing designs.
**Table S12**. MSE values for the M3 model for fyld under different sparse testing designs.

Figure S1. Phenotypic variance partitioning for dm and fyld.

## Data Availability

The R scripts and the phenotypic and genomic datasets that were used to perform the analysis are publicly available on the figshare repository: https://doi.org/10.6084/m9.figshare.26106526.
