# Supplementary material for: Sparse testing designs for optimizing resource allocation in multi‐environment cassava breeding trials
Source: Plant Genome. 2025 Feb 6;18(1):e20558. doi: 10.1002/tpg2.20558 (PMC11800058; doi:10.1002/tpg2.20558)
Supplement: Supplementary file 1 [file TPG2-18-e20558-s001.docx]

Table S 1. Predictive ability of the M1 model for dm under different sparse testing designs

|  | 87/0 | 77/10 | 67/20 | 57/30 | 47/40 | 37/50 | 27/60 | 17/70 | 7/80 | 0/87 |
| --- | --- | --- | --- | --- | --- | --- | --- | --- | --- | --- |
| 87 | 0.49 | 0.47 | 0.43 | 0.40 | 0.35 | 0.33 | 0.26 | 0.21 | 0.15 | 0.01 |
| 77 | NA | 0.46 | 0.42 | 0.40 | 0.35 | 0.32 | 0.26 | 0.21 | 0.14 | -0.01 |
| 67 | NA | NA | 0.41 | 0.39 | 0.34 | 0.31 | 0.25 | 0.21 | 0.14 | -0.02 |
| 57 | NA | NA | NA | 0.38 | 0.34 | 0.31 | 0.24 | 0.19 | 0.14 | 0.00 |
| 47 | NA | NA | NA | NA | 0.33 | 0.30 | 0.24 | 0.20 | 0.12 | -0.01 |

Table S 2. Predictive ability of the M2 model for dm under different sparse testing designs.

|  | 87/0 | 77/10 | 67/20 | 57/30 | 47/40 | 37/50 | 27/60 | 17/70 | 7/80 | 0/87 |
| --- | --- | --- | --- | --- | --- | --- | --- | --- | --- | --- |
| 87 | 0.51 | 0.51 | 0.49 | 0.46 | 0.43 | 0.42 | 0.36 | 0.33 | 0.28 | 0.25 |
| 77 | NA | 0.50 | 0.48 | 0.46 | 0.42 | 0.41 | 0.35 | 0.32 | 0.26 | 0.24 |
| 67 | NA | NA | 0.46 | 0.45 | 0.40 | 0.39 | 0.34 | 0.30 | 0.25 | 0.23 |
| 57 | NA | NA | NA | 0.44 | 0.40 | 0.39 | 0.33 | 0.28 | 0.24 | 0.21 |
| 47 | NA | NA | NA | NA | 0.38 | 0.38 | 0.32 | 0.27 | 0.23 | 0.19 |

Table S 3. Predictive ability of the M3 model for dm under different sparse testing designs.

|  | 87/0 | 77/10 | 67/20 | 57/30 | 47/40 | 37/50 | 27/60 | 17/70 | 7/80 | 0/87 |
| --- | --- | --- | --- | --- | --- | --- | --- | --- | --- | --- |
| 87 | 0.53 | 0.52 | 0.50 | 0.48 | 0.44 | 0.43 | 0.38 | 0.35 | 0.30 | 0.27 |
| 77 | NA | 0.52 | 0.49 | 0.47 | 0.43 | 0.42 | 0.38 | 0.34 | 0.29 | 0.26 |
| 67 | NA | NA | 0.48 | 0.46 | 0.41 | 0.41 | 0.36 | 0.33 | 0.27 | 0.25 |
| 57 | NA | NA | NA | 0.45 | 0.40 | 0.40 | 0.34 | 0.30 | 0.25 | 0.21 |
| 47 | NA | NA | NA | NA | 0.39 | 0.38 | 0.33 | 0.28 | 0.24 | 0.20 |

Table S 4. Predictive ability of the M1 model for fyld under different sparse testing designs

|  | 87/0 | 77/10 | 67/20 | 57/30 | 47/40 | 37/50 | 27/60 | 17/70 | 7/80 | 0/87 |
| --- | --- | --- | --- | --- | --- | --- | --- | --- | --- | --- |
| 87 | 0.44 | 0.41 | 0.38 | 0.35 | 0.33 | 0.29 | 0.24 | 0.20 | 0.14 | 0.02 |
| 77 | NA | 0.41 | 0.37 | 0.35 | 0.32 | 0.29 | 0.24 | 0.19 | 0.14 | 0.00 |
| 67 | NA | NA | 0.36 | 0.34 | 0.32 | 0.28 | 0.23 | 0.19 | 0.14 | -0.02 |
| 57 | NA | NA | NA | 0.33 | 0.32 | 0.28 | 0.23 | 0.19 | 0.15 | 0.01 |
| 47 | NA | NA | NA | NA | 0.31 | 0.27 | 0.22 | 0.18 | 0.13 | -0.02 |

Table S 5. Predictive ability of the M2 model for fyld under different sparse testing designs.

|  | 87/0 | 77/10 | 67/20 | 57/30 | 47/40 | 37/50 | 27/60 | 17/70 | 7/80 | 0/87 |
| --- | --- | --- | --- | --- | --- | --- | --- | --- | --- | --- |
| 87 | 0.44 | 0.43 | 0.40 | 0.38 | 0.35 | 0.33 | 0.27 | 0.24 | 0.21 | 0.15 |
| 77 | NA | 0.42 | 0.39 | 0.38 | 0.34 | 0.32 | 0.27 | 0.24 | 0.21 | 0.14 |
| 67 | NA | NA | 0.38 | 0.36 | 0.34 | 0.31 | 0.27 | 0.23 | 0.20 | 0.14 |
| 57 | NA | NA | NA | 0.35 | 0.34 | 0.30 | 0.26 | 0.22 | 0.20 | 0.13 |
| 47 | NA | NA | NA | NA | 0.32 | 0.29 | 0.25 | 0.22 | 0.18 | 0.13 |

Table S 6. Predictive ability of the M3 model for fyld under different sparse testing designs.

|  | 87/0 | 77/10 | 67/20 | 57/30 | 47/40 | 37/50 | 27/60 | 17/70 | 7/80 | 0/87 |
| --- | --- | --- | --- | --- | --- | --- | --- | --- | --- | --- |
| 87 | 0.47 | 0.45 | 0.42 | 0.41 | 0.38 | 0.35 | 0.31 | 0.27 | 0.24 | 0.20 |
| 77 | NA | 0.44 | 0.41 | 0.39 | 0.37 | 0.34 | 0.30 | 0.27 | 0.24 | 0.19 |
| 67 | NA | NA | 0.40 | 0.39 | 0.36 | 0.32 | 0.29 | 0.26 | 0.22 | 0.18 |
| 57 | NA | NA | NA | 0.38 | 0.35 | 0.32 | 0.28 | 0.25 | 0.23 | 0.16 |
| 47 | NA | NA | NA | NA | 0.34 | 0.30 | 0.26 | 0.24 | 0.20 | 0.14 |

Table S 7. MSE values of the M1 model for dm under different sparse testing designs.

|  | 87/0 | 77/10 | 67/20 | 57/30 | 47/40 | 37/50 | 27/60 | 17/70 | 7/80 | 0/87 |
| --- | --- | --- | --- | --- | --- | --- | --- | --- | --- | --- |
| 87 | 4.99 | 5.06 | 5.16 | 5.34 | 5.58 | 5.65 | 5.99 | 6.03 | 6.24 | 6.31 |
| 77 | NA | 5.23 | 5.27 | 5.35 | 5.62 | 5.67 | 5.97 | 6.04 | 6.21 | 6.39 |
| 67 | NA | NA | 5.57 | 5.46 | 5.67 | 5.75 | 6.07 | 6.05 | 6.30 | 6.27 |
| 57 | NA | NA | NA | 5.77 | 5.72 | 5.74 | 6.00 | 6.19 | 6.27 | 6.40 |
| 47 | NA | NA | NA | NA | 6.05 | 5.82 | 6.04 | 6.14 | 6.34 | 6.31 |

Table S 8. MSE) values of the M2 model for dm under different sparse testing designs.

|  | 87/0 | 77/10 | 67/20 | 57/30 | 47/40 | 37/50 | 27/60 | 17/70 | 7/80 | 0/87 |
| --- | --- | --- | --- | --- | --- | --- | --- | --- | --- | --- |
| 87 | 4.94 | 4.80 | 4.81 | 4.99 | 5.22 | 5.26 | 5.58 | 5.64 | 5.90 | 6.00 |
| 77 | NA | 5.07 | 4.92 | 5.01 | 5.28 | 5.30 | 5.57 | 5.70 | 5.92 | 6.08 |
| 67 | NA | NA | 5.25 | 5.11 | 5.38 | 5.40 | 5.70 | 5.75 | 6.03 | 5.99 |
| 57 | NA | NA | NA | 5.50 | 5.43 | 5.41 | 5.69 | 5.91 | 6.03 | 6.19 |
| 47 | NA | NA | NA | NA | 5.77 | 5.51 | 5.74 | 5.91 | 6.11 | 6.12 |

Table S 9. MSE values of the M3 model for dm under different sparse testing designs.

|  | 87/0 | 77/10 | 67/20 | 57/30 | 47/40 | 37/50 | 27/60 | 17/70 | 7/80 | 0/87 |
| --- | --- | --- | --- | --- | --- | --- | --- | --- | --- | --- |
| 87 | 4.56 | 4.67 | 4.72 | 4.88 | 5.15 | 5.18 | 5.46 | 5.53 | 5.81 | 5.89 |
| 77 | NA | 4.73 | 4.81 | 4.93 | 5.23 | 5.23 | 5.45 | 5.58 | 5.81 | 5.98 |
| 67 | NA | NA | 4.95 | 5.03 | 5.37 | 5.30 | 5.61 | 5.66 | 5.93 | 5.91 |
| 57 | NA | NA | NA | 5.17 | 5.42 | 5.35 | 5.63 | 5.86 | 6.01 | 6.14 |
| 47 | NA | NA | NA | NA | 5.55 | 5.50 | 5.72 | 5.87 | 6.05 | 6.07 |

Table S 10 MSE values of the M1 model for fyld under different sparse testing designs

|  | 87/0 | 77/10 | 67/20 | 57/30 | 47/40 | 37/50 | 27/60 | 17/70 | 7/80 | 0/87 |
| --- | --- | --- | --- | --- | --- | --- | --- | --- | --- | --- |
| 87 | 34.03 | 32.31 | 33.37 | 33.75 | 34.15 | 35.52 | 36.68 | 37.25 | 38.08 | 39.02 |
| 77 | NA | 34.71 | 33.57 | 34.32 | 34.24 | 35.37 | 36.76 | 37.43 | 38.26 | 39.51 |
| 67 | NA | NA | 36.10 | 34.68 | 34.78 | 35.54 | 36.87 | 37.65 | 38.03 | 39.19 |
| 57 | NA | NA | NA | 37.29 | 34.81 | 36.36 | 36.93 | 37.14 | 38.15 | 39.09 |
| 47 | NA | NA | NA | NA | 37.98 | 36.15 | 37.38 | 37.32 | 37.99 | 39.49 |

Table S 11. MSE values of the M2 model for fyld under different sparse testing designs.

|  | 87/0 | 77/10 | 67/20 | 57/30 | 47/40 | 37/50 | 27/60 | 17/70 | ‘7/80 | 0/87 |
| --- | --- | --- | --- | --- | --- | --- | --- | --- | --- | --- |
| 87 | 33.34 | 31.77 | 32.71 | 32.97 | 33.54 | 34.70 | 35.98 | 36.49 | 37.26 | 38.22 |
| 77 | NA | 33.56 | 32.90 | 33.55 | 33.60 | 34.64 | 36.10 | 36.60 | 37.49 | 38.82 |
| 67 | NA | NA | 34.55 | 33.88 | 34.19 | 34.85 | 36.19 | 37.02 | 37.33 | 38.52 |
| 57 | NA | NA | NA | 36.54 | 34.20 | 35.78 | 36.29 | 36.51 | 37.53 | 38.53 |
| 47 | NA | NA | NA | NA | 36.98 | 35.62 | 36.86 | 36.73 | 37.48 | 38.94 |

Table S 12. MSE values for the M3 model for fyld under different sparse testing designs.

|  | 87/0 | 77/10 | 67/20 | 57/30 | 47/40 | 37/50 | 27/60 | 17/70 | 7/80 | 0/87 |
| --- | --- | --- | --- | --- | --- | --- | --- | --- | --- | --- |
| 87 | 30.85 | 31.12 | 31.95 | 32.14 | 32.67 | 33.97 | 35.07 | 35.78 | 36.46 | 37.52 |
| 77 | NA | 31.38 | 32.40 | 32.92 | 32.95 | 34.00 | 35.34 | 35.84 | 36.70 | 38.11 |
| 67 | NA | NA | 32.73 | 33.20 | 33.69 | 34.45 | 35.58 | 36.45 | 36.73 | 37.81 |
| 57 | NA | NA | NA | 33.46 | 33.94 | 35.18 | 35.88 | 36.17 | 36.90 | 38.07 |
| 47 | NA | NA | NA | NA | 34.91 | 35.55 | 36.69 | 36.28 | 36.97 | 38.92 |
