# Supplementary material for: Sparse testing designs for optimizing resource allocation in multi‐environment cassava breeding trials
Source: Plant Genome. 2025 Feb 6;18(1):e20558. doi: 10.1002/tpg2.20558 (PMC11800058; doi:10.1002/tpg2.20558)
Supplement: Supplementary file 1 — Table S1. Predictive ability of the M1 model for dm under different sparse testing designs Table S2. Predictive ability of the M2 model for dm under different sparse testing designs. Table S3. Predictive ability of the M3 model for dm under different sparse testing designs. Table S4. Predictive ability of the M1 model for fyld under different sparse testing designs Table S5. Predictive ability of the M2 model for fyld under different sparse testing designs. Table S6. Predictive ability of the M3 model for fyld under different sparse testing designs. Table S7. MSE values of the M1 model for dm under different sparse testing designs. Table S8. MSE values of the M2 model for dm under different sparse testing designs. Table S9. MSE values of the M3 model for dm under different sparse testing designs. Table S10. MSE values of the M1 model for fyld under different sparse testing designs. Table S11. MSE values of the M2 model for fyld under different sparse testing designs. Table S12. MSE values for the M3 model for fyld under different sparse testing designs. [file TPG2-18-e20558-s001.docx]

Table S 1. Predictive ability of the M1 model for dm under different sparse testing designs

|  | 87/0 | 77/10 | 67/20 | 57/30 | 47/40 | 37/50 | 27/60 | 17/70 | 7/80 | 0/87 |
| --- | --- | --- | --- | --- | --- | --- | --- | --- | --- | --- |
| 87 | 0.49 | 0.47 | 0.43 | 0.40 | 0.35 | 0.33 | 0.26 | 0.21 | 0.15 | 0.01 |
| 77 | NA | 0.46 | 0.42 | 0.40 | 0.35 | 0.32 | 0.26 | 0.21 | 0.14 | -0.01 |
| 67 | NA | NA | 0.41 | 0.39 | 0.34 | 0.31 | 0.25 | 0.21 | 0.14 | -0.02 |
| 57 | NA | NA | NA | 0.38 | 0.34 | 0.31 | 0.24 | 0.19 | 0.14 | 0.00 |
| 47 | NA | NA | NA | NA | 0.33 | 0.30 | 0.24 | 0.20 | 0.12 | -0.01 |

Table S 2. Predictive ability of the M2 model for dm under different sparse testing designs.

|  | 87/0 | 77/10 | 67/20 | 57/30 | 47/40 | 37/50 | 27/60 | 17/70 | 7/80 | 0/87 |
| --- | --- | --- | --- | --- | --- | --- | --- | --- | --- | --- |
| 87 | 0.51 | 0.51 | 0.49 | 0.46 | 0.43 | 0.42 | 0.36 | 0.33 | 0.28 | 0.25 |
| 77 | NA | 0.50 | 0.48 | 0.46 | 0.42 | 0.41 | 0.35 | 0.32 | 0.26 | 0.24 |
| 67 | NA | NA | 0.46 | 0.45 | 0.40 | 0.39 | 0.34 | 0.30 | 0.25 | 0.23 |
| 57 | NA | NA | NA | 0.44 | 0.40 | 0.39 | 0.33 | 0.28 | 0.24 | 0.21 |
| 47 | NA | NA | NA | NA | 0.38 | 0.38 | 0.32 | 0.27 | 0.23 | 0.19 |

Table S 3. Predictive ability of the M3 model for dm under different sparse testing designs.

|  | 87/0 | 77/10 | 67/20 | 57/30 | 47/40 | 37/50 | 27/60 | 17/70 | 7/80 | 0/87 |
| --- | --- | --- | --- | --- | --- | --- | --- | --- | --- | --- |
| 87 | 0.53 | 0.52 | 0.50 | 0.48 | 0.44 | 0.43 | 0.38 | 0.35 | 0.30 | 0.27 |
| 77 | NA | 0.52 | 0.49 | 0.47 | 0.43 | 0.42 | 0.38 | 0.34 | 0.29 | 0.26 |
| 67 | NA | NA | 0.48 | 0.46 | 0.41 | 0.41 | 0.36 | 0.33 | 0.27 | 0.25 |
| 57 | NA | NA | NA | 0.45 | 0.40 | 0.40 | 0.34 | 0.30 | 0.25 | 0.21 |
| 47 | NA | NA | NA | NA | 0.39 | 0.38 | 0.33 | 0.28 | 0.24 | 0.20 |

Table S 4. Predictive ability of the M1 model for fyld under different sparse testing designs

|  | 87/0 | 77/10 | 67/20 | 57/30 | 47/40 | 37/50 | 27/60 | 17/70 | 7/80 | 0/87 |
| --- | --- | --- | --- | --- | --- | --- | --- | --- | --- | --- |
| 87 | 0.44 | 0.41 | 0.38 | 0.35 | 0.33 | 0.29 | 0.24 | 0.20 | 0.14 | 0.02 |
| 77 | NA | 0.41 | 0.37 | 0.35 | 0.32 | 0.29 | 0.24 | 0.19 | 0.14 | 0.00 |
| 67 | NA | NA | 0.36 | 0.34 | 0.32 | 0.28 | 0.23 | 0.19 | 0.14 | -0.02 |
| 57 | NA | NA | NA | 0.33 | 0.32 | 0.28 | 0.23 | 0.19 | 0.15 | 0.01 |
| 47 | NA | NA | NA | NA | 0.31 | 0.27 | 0.22 | 0.18 | 0.13 | -0.02 |

Table S 5. Predictive ability of the M2 model for fyld under different sparse testing designs.

|  | 87/0 | 77/10 | 67/20 | 57/30 | 47/40 | 37/50 | 27/60 | 17/70 | 7/80 | 0/87 |
| --- | --- | --- | --- | --- | --- | --- | --- | --- | --- | --- |
| 87 | 0.44 | 0.43 | 0.40 | 0.38 | 0.35 | 0.33 | 0.27 | 0.24 | 0.21 | 0.15 |
| 77 | NA | 0.42 | 0.39 | 0.38 | 0.34 | 0.32 | 0.27 | 0.24 | 0.21 | 0.14 |
| 67 | NA | NA | 0.38 | 0.36 | 0.34 | 0.31 | 0.27 | 0.23 | 0.20 | 0.14 |
| 57 | NA | NA | NA | 0.35 | 0.34 | 0.30 | 0.26 | 0.22 | 0.20 | 0.13 |
| 47 | NA | NA | NA | NA | 0.32 | 0.29 | 0.25 | 0.22 | 0.18 | 0.13 |

Table S 6. Predictive ability of the M3 model for fyld under different sparse testing designs.

|  | 87/0 | 77/10 | 67/20 | 57/30 | 47/40 | 37/50 | 27/60 | 17/70 | 7/80 | 0/87 |
| --- | --- | --- | --- | --- | --- | --- | --- | --- | --- | --- |
| 87 | 0.47 | 0.45 | 0.42 | 0.41 | 0.38 | 0.35 | 0.31 | 0.27 | 0.24 | 0.20 |
| 77 | NA | 0.44 | 0.41 | 0.39 | 0.37 | 0.34 | 0.30 | 0.27 | 0.24 | 0.19 |
| 67 | NA | NA | 0.40 | 0.39 | 0.36 | 0.32 | 0.29 | 0.26 | 0.22 | 0.18 |
| 57 | NA | NA | NA | 0.38 | 0.35 | 0.32 | 0.28 | 0.25 | 0.23 | 0.16 |
| 47 | NA | NA | NA | NA | 0.34 | 0.30 | 0.26 | 0.24 | 0.20 | 0.14 |

Table S 7. MSE values of the M1 model for dm under different sparse testing designs.

|  | 87/0 | 77/10 | 67/20 | 57/30 | 47/40 | 37/50 | 27/60 | 17/70 | 7/80 | 0/87 |
| --- | --- | --- | --- | --- | --- | --- | --- | --- | --- | --- |
| 87 | 4.99 | 5.06 | 5.16 | 5.34 | 5.58 | 5.65 | 5.99 | 6.03 | 6.24 | 6.31 |
| 77 | NA | 5.23 | 5.27 | 5.35 | 5.62 | 5.67 | 5.97 | 6.04 | 6.21 | 6.39 |
| 67 | NA | NA | 5.57 | 5.46 | 5.67 | 5.75 | 6.07 | 6.05 | 6.30 | 6.27 |
| 57 | NA | NA | NA | 5.77 | 5.72 | 5.74 | 6.00 | 6.19 | 6.27 | 6.40 |
| 47 | NA | NA | NA | NA | 6.05 | 5.82 | 6.04 | 6.14 | 6.34 | 6.31 |

Table S 8. MSE) values of the M2 model for dm under different sparse testing designs.

|  | 87/0 | 77/10 | 67/20 | 57/30 | 47/40 | 37/50 | 27/60 | 17/70 | 7/80 | 0/87 |
| --- | --- | --- | --- | --- | --- | --- | --- | --- | --- | --- |
| 87 | 4.94 | 4.80 | 4.81 | 4.99 | 5.22 | 5.26 | 5.58 | 5.64 | 5.90 | 6.00 |
| 77 | NA | 5.07 | 4.92 | 5.01 | 5.28 | 5.30 | 5.57 | 5.70 | 5.92 | 6.08 |
| 67 | NA | NA | 5.25 | 5.11 | 5.38 | 5.40 | 5.70 | 5.75 | 6.03 | 5.99 |
| 57 | NA | NA | NA | 5.50 | 5.43 | 5.41 | 5.69 | 5.91 | 6.03 | 6.19 |
| 47 | NA | NA | NA | NA | 5.77 | 5.51 | 5.74 | 5.91 | 6.11 | 6.12 |

Table S 9. MSE values of the M3 model for dm under different sparse testing designs.

|  | 87/0 | 77/10 | 67/20 | 57/30 | 47/40 | 37/50 | 27/60 | 17/70 | 7/80 | 0/87 |
| --- | --- | --- | --- | --- | --- | --- | --- | --- | --- | --- |
| 87 | 4.56 | 4.67 | 4.72 | 4.88 | 5.15 | 5.18 | 5.46 | 5.53 | 5.81 | 5.89 |
| 77 | NA | 4.73 | 4.81 | 4.93 | 5.23 | 5.23 | 5.45 | 5.58 | 5.81 | 5.98 |
| 67 | NA | NA | 4.95 | 5.03 | 5.37 | 5.30 | 5.61 | 5.66 | 5.93 | 5.91 |
| 57 | NA | NA | NA | 5.17 | 5.42 | 5.35 | 5.63 | 5.86 | 6.01 | 6.14 |
| 47 | NA | NA | NA | NA | 5.55 | 5.50 | 5.72 | 5.87 | 6.05 | 6.07 |

Table S 10 MSE values of the M1 model for fyld under different sparse testing designs

|  | 87/0 | 77/10 | 67/20 | 57/30 | 47/40 | 37/50 | 27/60 | 17/70 | 7/80 | 0/87 |
| --- | --- | --- | --- | --- | --- | --- | --- | --- | --- | --- |
| 87 | 34.03 | 32.31 | 33.37 | 33.75 | 34.15 | 35.52 | 36.68 | 37.25 | 38.08 | 39.02 |
| 77 | NA | 34.71 | 33.57 | 34.32 | 34.24 | 35.37 | 36.76 | 37.43 | 38.26 | 39.51 |
| 67 | NA | NA | 36.10 | 34.68 | 34.78 | 35.54 | 36.87 | 37.65 | 38.03 | 39.19 |
| 57 | NA | NA | NA | 37.29 | 34.81 | 36.36 | 36.93 | 37.14 | 38.15 | 39.09 |
| 47 | NA | NA | NA | NA | 37.98 | 36.15 | 37.38 | 37.32 | 37.99 | 39.49 |

Table S 11. MSE values of the M2 model for fyld under different sparse testing designs.

|  | 87/0 | 77/10 | 67/20 | 57/30 | 47/40 | 37/50 | 27/60 | 17/70 | ‘7/80 | 0/87 |
| --- | --- | --- | --- | --- | --- | --- | --- | --- | --- | --- |
| 87 | 33.34 | 31.77 | 32.71 | 32.97 | 33.54 | 34.70 | 35.98 | 36.49 | 37.26 | 38.22 |
| 77 | NA | 33.56 | 32.90 | 33.55 | 33.60 | 34.64 | 36.10 | 36.60 | 37.49 | 38.82 |
| 67 | NA | NA | 34.55 | 33.88 | 34.19 | 34.85 | 36.19 | 37.02 | 37.33 | 38.52 |
| 57 | NA | NA | NA | 36.54 | 34.20 | 35.78 | 36.29 | 36.51 | 37.53 | 38.53 |
| 47 | NA | NA | NA | NA | 36.98 | 35.62 | 36.86 | 36.73 | 37.48 | 38.94 |

Table S 12. MSE values for the M3 model for fyld under different sparse testing designs.

|  | 87/0 | 77/10 | 67/20 | 57/30 | 47/40 | 37/50 | 27/60 | 17/70 | 7/80 | 0/87 |
| --- | --- | --- | --- | --- | --- | --- | --- | --- | --- | --- |
| 87 | 30.85 | 31.12 | 31.95 | 32.14 | 32.67 | 33.97 | 35.07 | 35.78 | 36.46 | 37.52 |
| 77 | NA | 31.38 | 32.40 | 32.92 | 32.95 | 34.00 | 35.34 | 35.84 | 36.70 | 38.11 |
| 67 | NA | NA | 32.73 | 33.20 | 33.69 | 34.45 | 35.58 | 36.45 | 36.73 | 37.81 |
| 57 | NA | NA | NA | 33.46 | 33.94 | 35.18 | 35.88 | 36.17 | 36.90 | 38.07 |
| 47 | NA | NA | NA | NA | 34.91 | 35.55 | 36.69 | 36.28 | 36.97 | 38.92 |
