# Supplementary material for: Sparse testing designs for optimizing resource allocation in multi‐environment cassava breeding trials
Source: Plant Genome. 2025 Feb 6;18(1):e20558. doi: 10.1002/tpg2.20558 (PMC11800058; doi:10.1002/tpg2.20558)
Supplement: Supplementary file 2 — Figure S1. Phenotypic variance partitioning for dm and fyld. [file TPG2-18-e20558-s002.docx]

Figure S1. Phenotypic variance partitioning for dm and fyld.
